# Influence of Artichoke Antioxidant Activity in Their Susceptibility to Suffer Frost Injury

**DOI:** 10.3390/antiox12111960

**Published:** 2023-11-02

**Authors:** Marina Giménez-Berenguer, María Gutiérrez-Pozo, Vicente Serna-Escolano, María José Giménez, Pedro Javier Zapata

**Affiliations:** Department of Food Technology, Escuela Politécnica Superior de Orihuela, University Miguel Hernández, Ctra. Beniel km. 3.2, 03312 Alicante, Spain; marina.gimenezb@umh.es (M.G.-B.); maria.gutierrezp@umh.es (M.G.-P.); vserna@umh.es (V.S.-E.); pedrojzapata@umh.es (P.J.Z.)

**Keywords:** artichoke head order, frostbite, total phenolic content, size, weight

## Abstract

In the northern hemisphere countries, artichoke harvest occurs in winter months; consequently, they are exposed to cold temperatures. This can lead to frost injury, such as triggering the blistering of the cuticle and detachment of outer bracts, which eventually could display brown or black discolouration. This can cause major economic and production losses. As far as we know, no literature is available about this problem in artichokes. Thus, the main aim of this study was to evaluate the effect of total phenolic content and the antioxidant potential of ‘Blanca de Tudela’ artichokes in their capacity to tolerate frost injury when they are exposed to low temperatures. Several factors were analysed, including floral head order, weight and size of artichokes, total phenolic content, phenolic profile and total antioxidant activity. Results showed that tertiary heads, which are the smallest in size, exhibited a greater amount of total phenolic content and antioxidant activity. As a result, these characteristics offered enhanced protection to the artichoke against frosting temperatures. In contrast, the largest artichokes, especially the primary heads, were more susceptible to suffer frostbite. Therefore, artichokes with robust antioxidant systems, characterized by elevated phenolic content, are crucial to reduce their susceptibility to frost injury.

## 1. Introduction

Spain is the third largest artichoke producing country in the world, only surpassed by Italy and Egypt [1]. These countries are located in the northern hemisphere and the harvesting schedule of artichokes typically goes from October to May. Additionally, the south of Spain presents a continuous production of artichokes during the winter months resulting in different harvest times, even in the coldest months of the year, January and February. In particular, the most cultivated variety in Spain, ‘Blanca de Tudela’, has the unique characteristic of presenting two harvest periods. The initial harvest occurs during the autumn–winter season, known as an early production, while the second harvest occurs in the spring season, referred to as a late production [2]. During the early production months, low temperatures and frost climatic conditions can occur, leading to frost injury or frostbite.

Frost stress produces plant cell injury due to the accumulation of reactive oxygen species (ROS) in plant cells with consequent oxidative damage [3]. However, the incidence of frost injury depends on different factors, including the sensitivity of the cultivar, the developmental stage, as well as the severity, duration, and frequency of frost conditions [4]. When artichoke inflorescences and leaves are exposed to temperatures below 0 °C, they can already begin to suffer physiological changes and damage. Frost injury in artichokes begins at −1.2 °C, resulting in the blistering of the cuticle, bronzing and detachment of the outer bracts. If the temperature drops further than −4 °C, the damage intensifies, causing the bracts to become water-soaked, showing necrosis and eventually brown or black spots, particularly affecting the heart of the artichoke [5,6].

Frost is a factor that causes important economic losses due to the reduction of fruit quality and yield in different crops. Frost injury has been studied in different crops such as faba bean [7], avocados [8], kale [9], olive tree [10], wheat [11] and lettuce [12], among others. Frost damage occurred in all parts of the faba bean plant, but the tolerance was higher in pods, followed by stems, with the flowers being the most susceptible part [7]. Olive trees are susceptible to frost damage, especially in the first years of planting, being the main symptoms observed in the aerial parts of the plant and resulting in shoot tip necrosis, leaf fall and bark wounding [10]. However, frost injury has never been evaluated in artichokes.

Artichokes are rich in bioactive compounds with high antioxidant activity and are considered a functional food beneficial to consumer health [13]. Secondary metabolites, such as phenols, are produced in plants under normal environmental conditions for signalling and metabolism. They play a key role in the regulation of development and tolerance mechanisms against biotic and abiotic stresses [14]. Additionally, when plants are exposed to temperatures below 0 °C, the biosynthesis pathway of antioxidant compounds is enhanced, and these metabolites prevent plants from the phytotoxic effects of extreme temperatures. This prevents the oxidation of important proteins and lipids of the cell membrane produced by the ROS and preserves its integrity [15]. Two kinds of molecules are involved in the antioxidant system of plants known by their origin as enzymatic (catalase, ascorbate peroxidase or superoxide dismutase) and non-enzymatic (ascorbic acid, phenolic compounds or flavonoids) [16]. In this sense, phenolics and flavonoids are considered to be excellent electron donors due to the presence of hydroxyl groups, which are directly related to their antioxidant properties [17,18,19,20]. Moreover, some phenolic compounds, such as hydroxycinnamic acids and luteolin derivates, have been found to stimulate the production of endogenous antioxidant molecules within cells [21]. Several studies have reported that phenolic compounds are capable of neutralising free radicals, breaking down peroxides, deactivating metal ions, scavenging oxygen in biological systems, and preventing oxidative reactions [22,23,24].

The qualitative and quantitative profile of artichokes can be influenced by several preharvest factors such as harvest date, genotype, agronomic management and environmental conditions [13,25,26,27,28,29]. Moreover, previous studies have reported that total phenolic content in different artichoke cultivars are highly influenced by the flower head order. Tertiary head orders presented the highest individual phenolic content, followed by the secondary and main heads, with this effect being cultivar-dependent [2,30]. Therefore, the aim of this research was to evaluate the effect of total phenolic content, and, therefore, the antioxidant potential of ‘Blanca de Tudela’ artichokes in their capacity to tolerate frost injury when they are exposed to low temperatures.

## 2. Materials and Methods

### 2.1. Plant Material and Experimental Design

The experiment was carried out in an experimental plot of Miguel Hernández University (Orihuela, Southeast Spain; 38°06′63.52″ N, −0°988′09.29″ W). ‘Blanca de Tudela’ cultivar was studied along the developmental cycle (autumn 2022 to spring 2023). Thereby, 300 offshoots from first-year artichoke plants were planted in a planting frame of 0.8 m × 1.2 m at the beginning of August. Crop management was performed according to standard commercial agronomic practices. Along the developmental cycle, insecticides, fungicides and fertilizers composed of 250 kg N, 120 kg P_2_O_5_, and 300 kg K_2_O per ha were applied using a drip irrigation system.

Even though the growing area has a Mediterranean climate, characterized by mild and humid winters and hot and rainless summers, January, the month before harvest, was characterized by having an absolute maximum and minimum temperature of 23.4 °C and −1.3 °C, respectively, and an average relative humidity of 57% (weather station 7244X, Orihuela-Desamparados, 38°04′04″ N, 00°58′53″ W). Considering that the minimum temperature dates when frost injury occurred (≤0.0 °C) were 29 and 30 January, the weather conditions by hours are presented in detail in Table S1. The average temperature and relative humidity were 2 and 68% on day 29, and 8 and 67% on day 30, respectively. Only one day of rainfall was recorded in January, with values of total precipitation of 0.2 mm.

**Figure 1:**
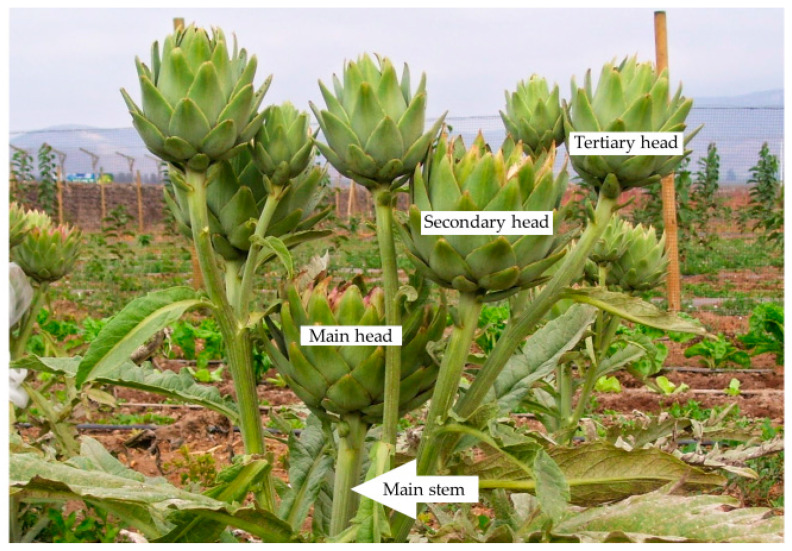
Flower head order disposal in artichoke plant.

All artichokes were harvested with a 15–20 cm floral stem on the 1 February 2023 according to the commercial criteria, based on head size and morphology, when they were tightly closed and firm. During harvest, they were classified according to the flower head order: main, secondary and tertiary heads. Main artichokes emerge from the central stem of the plant and are the ones that reach the lowest height. Its ramifications give rise to the secondary artichokes with medium height. Tertiary heads grow up from the stem of the secondary ones and are the highest ones (Figure 1) [31].

### 2.2. Frost Injury Evaluation

**Figure 2:**
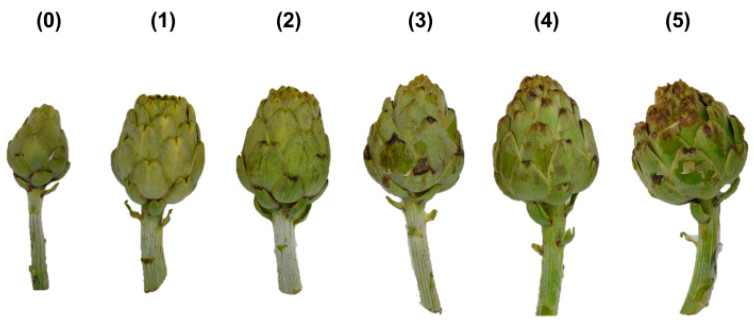
Frost injury scale: (**0**) no appreciable frost injury; (**1**) slight frost injury; (**2**) light frost injury; (**3**) moderated frost injury; (**4**) severe frost injury; (**5**) severe frost injury with dehydrations in external bracts.

A total of 330 artichokes were harvested and separated according to their flower head order (main, secondary and tertiary). Then, artichokes were transported to the laboratory where analytical determinations and an evaluation of the frost injury were performed. Each artichoke of these three groups was classified individually depending on the grade of frostbite presented (from 0—no frost injury to 5—severe frost injury) using a six-grade hedonic scale based on the severity of frost injury symptoms (Figure 2): 0 = no appreciable frost injury; 1 = slight frost injury; 2 = light frost injury with the extern bracts turning slightly brown in the ends; 3 = moderated frost injury with browner bracts ends; 4 = severe frost injury with greater brown spots; 5 = severe frost injury with greater brown spots and the presence of dehydration in the external bracts. The hedonic scale was performed with photographs taken by the authors.

### 2.3. Analytical Determinations

#### 2.3.1. Average Weight of Artichokes

All artichokes were weighed with 15–20 cm of the floral stem using a Radwag WLC 2/A2 Precision Balance (Radwag Wagi Elektroniczne, Radom, Poland) with accuracy to two decimal places. The average flower head weight was expressed as the mean ± standard error (SE) in grams (g).

#### 2.3.2. Size of Artichokes

Artichokes length, diameter and steam thickness were also measured with a precision digital calliper (Digimatic Caliper CD-P15K, Mitutoyo Corporation, Kanagawa, Japan) with accuracy to two decimal places. The average flower head size was expressed as the mean ± SE in millimetres (mm).

#### 2.3.3. Identification and Quantification of Individual Phenolic Compounds

Phenolic extraction was performed according to Giménez et al. [30]. Briefly, 5 g of the edible fraction (internal bracts and receptacle) was ground with 15 mL of a water/methanol solution (2:8) containing 2 mM NaF. This mixture was processed using an Ultra-Turrax^®^ (IKA TP 18, Staufen, Germany) for 2 min, followed by centrifugation at 10,000× *g* for 15 min at 4 °C. The identification and quantification of individual phenolics was carried out using both DAD-ESI/MSn (Bruker Daltonics Ultra HCT-ESI Ion Trap, Bremen, Germany) and RP-HPLC-DAD (Agilent HPLC 1200 Infinity series, Agilent Technologies, Waldbronn, Germany) systems. The hydroxycinnamic acids and luteolin derivatives were quantified at 320 and 360 nm using a calibration curve of two standards, 5-*O*-caffeoylquinic acid and 3-luteolin-*O*-rutinoside (Sigma Aldrich, Darmstadt, Germany), respectively [30]. The total phenolic content (TPC) was calculated by summing up the concentrations of individual polyphenols. The results were expressed as g kg^−1^ of fresh weight (FW) and represented the average ± SE of 10 frosted (affected by severe frost injury: grades 4–5) artichokes and 10 non-frosted (grade 0) artichokes, respectively.

#### 2.3.4. Total Antioxidant Activity

The quantification of total antioxidant activity (TAA) was conducted following the protocol described by Giménez et al. [30]. Briefly, for each individual artichoke, 2 g of the edible portion was homogenized with 10 mL of Na-phosphate buffer (50 mM, pH 7.8) and 5 mL of ethyl acetate for 2 min using an Ultra-Turrax^®^ (IKA TP 18, Staufen, Germany). Subsequently, the homogenate was centrifuged at 10,000× *g* for 15 min at 4 °C. The quantification of TAA was carried out from the sum of both hydrophilic and lipophilic compounds in the same extraction. The determination was performed by monitoring the reaction of ABTS+ radicals generated from the enzymatic system consisting of 2,2′-azino-bis-(3-ethylbenzothiazoline-6-sulfonic acid) diammonium salt (ABTS), horse radish peroxidase enzyme (HRP), and the oxidant substrate (hydrogen peroxide). The absorbance was measured at 730 nm. Results were expressed as the equivalent of Trolox (6-hydroxy-2,5,7,8-tetramethylchroman-2-carboxylic acid) per kilogram of fresh weight (g equivalent of Trolox kg^−1^ FW). The values were the mean ± SE of 10 frosted (affected by severe frost injury: grades 4–5) artichokes and 10 non-frosted (grade 0) artichokes, respectively.

### 2.4. Statistical Analysis

Data was subjected to the analysis of variance (ANOVA). Mean comparisons were performed using a multiple-range test (HSD Duncan’s test) to detect significant differences (*p* < 0.05). ANOVA assumptions were tested and found to be valid for this dataset. All analyses were performed with SPSS software package v. 20 for Windows (IBM Corp., Armonk, NY, USA).

## 3. Results

### 3.1. Influence of Artichoke Flower Head Order, Weight and Size of Artichokes on Frost Injury

**Figure 3:**
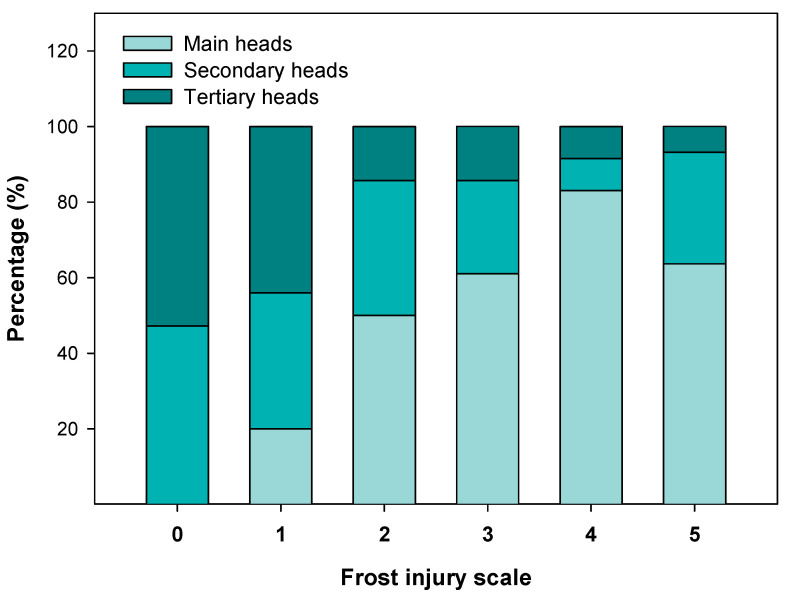
Percentage of artichokes affected by frost injury attending to the flower head order in every frost injury grade.

Artichokes were separated according to their flower head order (main, secondary and tertiary) at harvest, and then, in the laboratory, each artichoke of these three groups was classified individually depending on the grade of frostbite presented (from 0—no frost injury to 5—severe frost injury). The percentages of main, secondary and tertiary artichoke heads in each frost injury grade are presented in Figure 3.

Artichokes that did not have any frost injury (grade 0) were mainly tertiary heads, (52.78%), followed by secondary ones with 47.22%. Nevertheless, no main artichokes were found without frost injury. Additionally, main artichokes were the most affected with a higher frost injury (grades 4 and 5) with an average percentage of 73.34%, after a 19.01% of secondary heads and 7.65% of tertiary heads (Figure 3).

**Figure 4:**
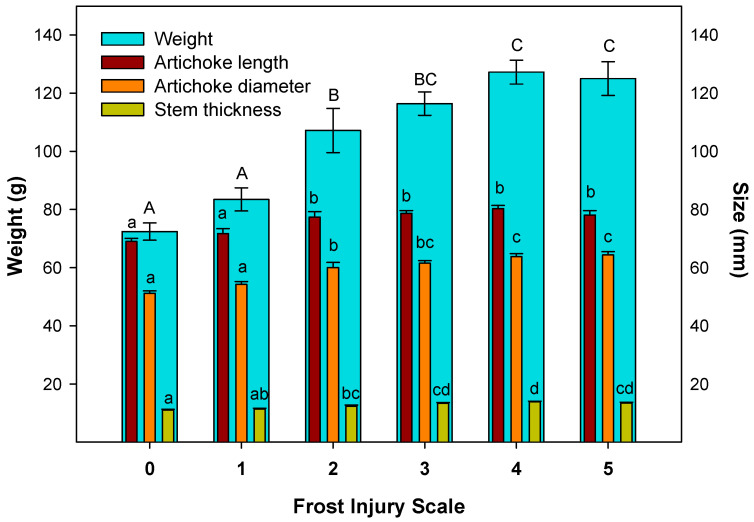
Average weight (g) and size (mm) of ‘Blanca de Tudela’ artichokes in each frost injury grade. Results were expressed as mean values ± SE. Different letters show significant differences (*p* < 0.05 according to HSD Duncan’s test) in weight (capital letters) and size (lowercase letters) between frost injury grades.

Furthermore, the correlation between the weight and size of artichoke heads and their susceptibility to be affected with frost injury was studied (Figure 4). The smallest artichokes, also classified as tertiary heads, were found on the lowest frost injury scales (0 and 1). Medium artichokes (secondary heads) presented lower frost injury (grades 2 and 3) than the bigger ones (main heads), which showed the highest frost injury (grades 4 and 5).

In grade 0, artichokes with no frost injury had an average weight, artichoke length, equatorial diameter and stem thickness of 72.40 ± 2.96 g, 68.98 ± 1.13 mm, 51.17 ± 0.85 mm and 11.02 ± 0.30 mm, respectively. However, the most affected artichokes, grades 4 and 5, presented significant differences (*p* < 0.05) compared to artichokes with lower frost grades, with the average weight, artichoke length, equatorial diameter and stem thickness of 126.11 ± 4.95 g, 79.18 ± 1.31 mm, 64.10 ± 1.05 mm and 13.63 ± 0. 31 mm, respectively (Figure 4).

Therefore, there was a relationship between the susceptibility to frost injury and the size of the flower head order. The difference involving the weight of grade 0 and grades 4 and 5 artichokes was almost a two-fold increase. Meanwhile, parameters such as length, equatorial diameter and stem thickness increased by 12.88%, 20.16% and 19.14%, respectively. Thus, there was a close relation between the weight and size of artichokes and the flower head’s order.

### 3.2. Influence of Phenolic Content and Different Phenolic Profiles on Frost Injury

**Figure 5:**
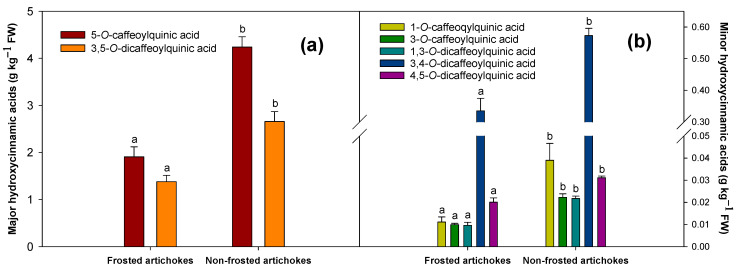
Hydroxycinnamic profile: (**a**) Major hydroxycinnamic acids; (**b**) Minor hydroxycinnamic acids) and content (g kg^−1^ FW) for ‘Blanca de Tudela’ frosted (grades 4 and 5) and non-frosted artichokes (grade 0). Results were expressed as mean values ± SE. Different letters show significant differences (*p* < 0.05 according to HSD Duncan’s test) between frosted and non-frosted artichokes for each individual phenolic compound.

The phenolic profile was evaluated for frosted (grades 4 and 5) and non-frosted artichokes (grade 0). Seven hydroxycinnamic acids were identified in both types of artichokes, being 5-*O*-caffeoylquinic acid and 3,5-*O*-dicaffeoylquinic acid the major ones (Figure 5a). The five minor hydroxycinnamic acids found, attending to their concentration, were 3,4-*O*-dicaffeoylquinic acid, 1-*O*-caffeoqylquinic acid, 4,5-*O*-dicaffeoylquinic acid, 3-*O*-caffeoylquinic acid and 1,3-*O*-dicaffeoylquinic acid (Figure 5b).

Results showed that all hydroxycinnamic acids were detected in higher concentrations in non-frosted artichokes than in frosted ones. Particularly, a two-fold increase was observed on almost every compound identified, increasing the total hydroxycinnamic acid content by 51.58% (Figure 5a,b).

The concentration of 5-*O*-caffeoylquinic acid, the major phenolic compound, was 1.908 ± 0.211 g kg^−1^ FW for frosted artichokes and 4.239 ± 0.217 g kg^−1^ FW for non-frosted artichokes, showing an increase of 55%. Furthermore, for the second major phenolic compound, 3,5-*O*-dicaffeoylquinic, an increase of 48.18% (1.379 ± 0.136 and 2.658 ± 0.211 g kg^−1^ FW for frosted and non-frosted artichokes, respectively) was found (Figure 5a).

A similar tendency was observed for the minor hydroxycinnamic acids; in general, their concentration was significantly higher in non-frosted artichokes than the frosted ones (Figure 5b). The concentration of 1-*O*-caffeoqylquinic acid observed, which almost triplicated its content, had values of 0.011 ± 0.002 g kg^−1^ FW in frosted artichokes and 0.039 ± 0.008 g kg^−1^ FW in non-frosted ones (Figure 5b). This was the minor hydroxycinnamic acid which showed the highest increment, changing the importance of 1-*O*-caffeoqylquinic acid in non-frosted artichokes compared to frosted ones.

**Figure 6:**
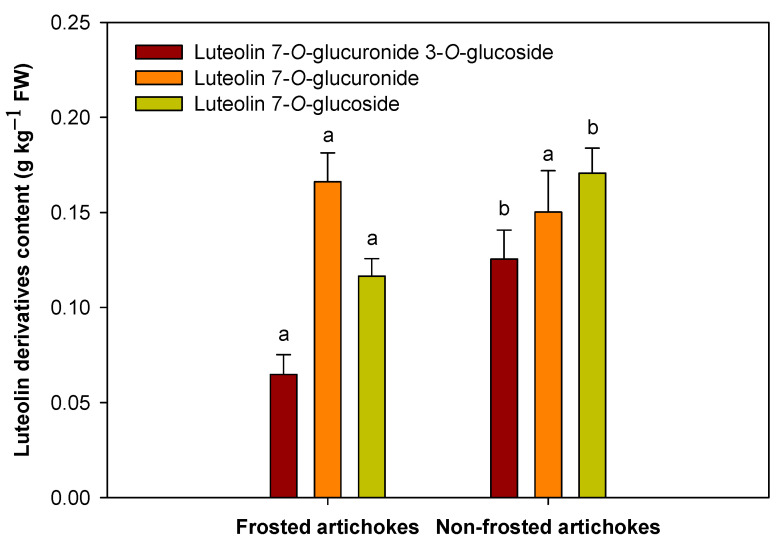
Luteolin profile and content (g kg^−1^ FW) for ‘Blanca de Tudela’ frosted (grades 4 and 5) and non-frosted artichokes (grade 0). Results were expressed as mean values ± SE. Different letters show significant differences (*p* < 0.05 according to HSD Duncan’s test) between frosted and non-frosted artichokes for luteolin derivatives content.

Moreover, three different luteolins were identified: Luteolin 7-*O*-glucoside was the major one, followed by Luteolin 7-*O*-glucuronide, and Luteolin 7-*O*-glucuronide 3-*O*-glucoside (Figure 6). Luteolin’s derivatives content showed a similar tendency as the hydroxycinnamic acids, being 22.15% higher in non-frosted artichokes than in frosted ones, except for Luteolin 7-*O*-glucuronide, which did not present any significant differences (*p* < 0.05).

Luteolin 7-*O*-glucoside was the major compound for the non-frosted artichokes, being 31.73% higher than in frosted ones, with a concentration of 0.116 ± 0.009 g and 0.170 ± 0.013 g kg^−1^ FW for frosted and non-frosted, respectively. Furthermore, Luteolin 7-*O*-glucuronide 3-*O*-glucoside showed the highest increase (48.40%) in non-frosted artichokes compared to frosted ones, despite being the minor luteolin (Figure 6).

### 3.3. Influence of Total Phenolic Content and Total Antioxidant Activity on Frost Injury

**Figure 7:**
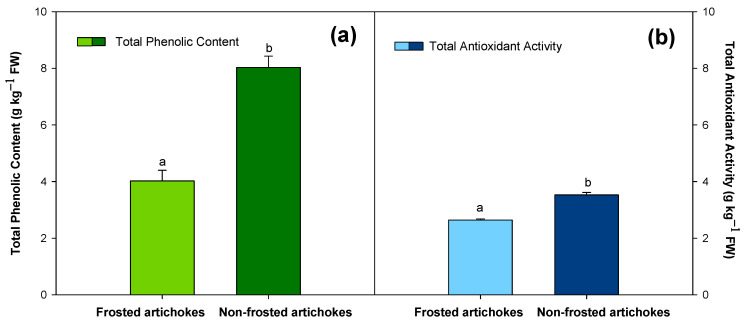
(**a**) Total phenolic content (g kg^−1^ FW) and (**b**) total antioxidant activity (g kg^−1^ FW) for ‘Blanca de Tudela’ frosted (grades 4 and 5) and non-frosted artichokes (grade 0). Results were expressed as mean values ± SE. Different letters show significant differences (*p* < 0.05 according to HSD Duncan’s test) between frosted and non-frosted artichokes for total antioxidant activity and total phenolic content.

The TAA and TPC, calculated as the sum of the individual phenolic compounds, were evaluated for frosted and non-frosted artichokes (Figure 7a,b). Results showed that the TPC was significantly higher (*p* < 0.05) in non-frosted artichokes compared to frosted ones, with an increase of 49.94% (4.020 ± 0.379 g and 8.031 ± 0.402 g kg^−1^ FW for frosted and not-frosted, respectively) (Figure 7a). Regarding TAA, it was 25.09% higher in non-frosted artichokes than in frosted ones with values of 2.644 ± 0.038 g kg^−1^ FW and 3.529 ± 0.085 g kg^−1^ FW, respectively (Figure 7b).

## 4. Discussion

The aim of this study was to understand the effect of the total phenolic and antioxidant content of ‘Blanca de Tudela’ artichokes in their susceptibility to suffer frost injury, helping to mitigate the consequent production and economic losses involved. Low temperatures can cause damage to all plants, but the mechanisms and type of injury could vary considerably [4]. In the present study, the percentage of artichokes affected by frost injury was evaluated. A high percentage of tertiary heads presented a frost injury grade of 0 and 1, showing less susceptibility to be affected by frost injury followed by the secondary heads. In contrast, main artichokes were more affected by frostbite, since a higher number of artichokes were classified with a higher injury grade. Even though grades 4 and 5 were both associated with the highest frost injury, differences between them were detected, and higher dehydration symptoms were observed in the last grade. Frost injury produced by ice is mainly related to the physical disruption of cellular structures and subsequent dehydration due to the increased water potential in the interior of the cells compared to the space between them where ice crystals are formed [32,33].

The flower order in plants determines the characteristics or physical parameters of artichokes [2]. The main artichokes tend to be the biggest, followed by the secondary heads, and tertiary artichokes being the smallest [2,25,30], in accordance with the results achieved in the present study. In this sense, artichoke is not the only vegetable where there is a differentiation based on its time of formation and position on the stem. A study on faba beans found that the weight of seeds in the lower position was higher than in the middle and upper positions [34]. Another study on pigeon peas showed that the early forming pods were usually heavier than those forming later [35].

The susceptibility of artichokes to suffer frost injury could be determined by their position on the plant. As previously mentioned, main artichokes are those that grow from the central stem of the plant and therefore could be more protected by the leaves. Lateral ramifications of the central stem led to secondary artichokes and from the stem of the secondary ones, tertiary heads were produced. Due to the morphology of the artichoke plant, secondary, and especially tertiary heads, were developed higher in the plant (Figure 1) [31], and they could be more exposed to climatic conditions, and therefore, may suffer more from frost injury. However, in this study, the opposite effect was observed, being that the main artichokes presented the highest grade of frost injury, followed by secondary and tertiary ones. Thus, the position of the artichoke in the plant did not present any effect on their susceptibility and grade of frost injury. The differences observed could be due to the role that bioactive content plays during biotic and abiotic stresses [14].

Artichoke heads are known to have high nutritional value, mainly due to their rich polyphenol content [26,36], including hydroxycinnamic acids and flavonoids, such as luteolins [2,27]. In the present study, individual phenolic content results showed that the seven hydroxycinnamic acids and the three luteolin derivatives analysed were higher in non-frosted artichokes compared to the frosted ones. This is the first study that describes that these changes in phenolic content influence the susceptibility of artichokes to suffer frost damage. The most abundant hydroxycinnamic acids quantified in ‘Blanca de Tudela’ cultivar were 5-*O*-caffeoylquinic acid (chlorogenic acid), followed by 3,5-di-*O*-caffeoylquinic acid, in agreement with previous studies performed on different artichoke cultivars [2,27,37,38,39,40]. In general, the hydroxycinnamic acids quantified were at least two-fold higher in non-frosted than in frosted artichokes. A similar trend was observed for hydroxycinnamic acids and for two of the three luteolins quantified. Luteolin 7-*O*-glucoside and luteolin 7-*O*-glucuronide 3-*O*-glucoside presented higher concentration for non-frosted artichokes compared to frosted ones, but no differences were found for Luteolin 7-*O*-glucuronide. However, there are few studies that have reported changes in the hydroxycinnamic acid and luteolin profile. The influence of artichoke head order (main, secondary and tertiary) on the phenolic content (hydroxycinnamic acid and luteolin content) has been previously described in different artichoke cultivars [2,25]. In general, hydroxycinnamic acid content varied among flower head orders, showing that tertiary heads had higher levels than the main or secondary heads for major and minor hydroxycinnamic acids; however, this tendency was cultivar-dependent. The total amount of each individual luteolin was significantly higher in tertiary flower head orders than secondary or main heads. In particular, luteolin-7-*O*-glucuronide was the major luteolin identified in all artichoke cultivars [2]. These results are in agreement with the results obtained for frosted artichokes; however, for non-frosted artichokes it was lut-7-*O*-glucoside the one presenting the greatest concentration.

The sum of individual phenolic content was represented as total phenolic content (TPC). Results showed that frosted artichokes presented lower TPC than non-frosted ones. A similar tendency was observed in the total antioxidant activity (TAA). Different authors have found a direct correlation between antioxidant capacity and total phenolic content [17,19,20,22]. The susceptibility of artichokes to suffer frost injury may be related to the differences in the TPC and TAA, which were also related to the flower head order. These findings suggest that artichokes with a higher phenolic content show better frost tolerance by enhancing their antioxidant defence systems. In this sense, tertiary artichokes presented the best aptitude to resist frostbite and frost conditions as their cryopathy incidence was lower. Although no studies have been carried out on the effect of frost injury in artichokes, these changes in the bioactive compounds according to climatic conditions have been previously studied in different crops such as avocado, kale leaves and lettuce plants [8,9,12]. On the one hand, it was reported that the antioxidant activity, mainly from a non-enzymatic source, such as the accumulation of phenolic compounds, was responsible for the increased tolerance to frost injury in avocados [8]. In that study, two different avocado cultivars were evaluated, ‘Ettinger’ and ‘Hass’. TPC and TAA were significantly higher in ‘Ettinger’ cultivar than in ‘Hass’, suggesting that the greater frost tolerance of the first cultivar was due to its greater content of antioxidant compounds [8]. The relation between greater TAA and better frost tolerance has also been previously described in pines in which it was observed that high levels of ROS elimination activities were associated with greater frost tolerance [41]. Another study found that the treatment of olive plants with salicylic acid (SA) increased their tolerance to freezing by increasing antioxidant enzyme activities and total phenolic content [42]. The defence of plants against stress is linked to the antioxidant capacity and higher levels of phenols play a protective role against stress damage [10], as the antioxidant potential often depends on the content of bioactive compounds [22].

It is well known that when plants are exposed to unfavourable conditions, an increase in ROS-scavenging activities is generally observed as a protection mechanism. In particular, low temperatures can induce the accumulation of antioxidant compounds in order to protect the cell membrane from breakdown and peroxidation. Redox reactions and turnover of ROS in cells can be carried out through non-enzymatic and enzymatic antioxidants that maintain ROS at a sub-lethal level [10]. The present study showed that the highest total phenolic content and total antioxidant activity of tertiary head artichokes increased their resistance to suffer frost injury or minimise its effects. Up to date, no literature is available related the susceptibility to frostbite in artichokes. This research is the first study where a relation between susceptibility to frost injury and total phenolic content, and the consequent total antioxidant activity attending to the flower head order, has been observed.

## 5. Conclusions

For the first time, the effect of total phenolic compounds on the susceptibility of ‘Blanca de Tudela’ artichoke to frost injury when they are exposed to low temperatures has been studied. Flower head order, along with weight, size, total phenolic content (TPC) and total antioxidant activity (TAA) played an important role in the susceptibility of artichokes to suffer frost injury. Tertiary head artichokes, also the smallest characterized, presented the highest TPC and TAA, which increased their resistance against frost temperatures and reduced their susceptibility to frost injury. On the contrary, the biggest artichokes, main heads, were more affected by adverse frost temperatures, presenting a higher frostbite incidence. In conclusion, artichokes with higher antioxidant systems related to their higher phenolic compounds would be less susceptible to suffer frost injury. Consequently, it is plausible that the application of elicitors, bio-stimulants, or other compounds capable of stimulating the antioxidant systems of the plant, could be used as a preventive measure against frost injury when extreme environmental conditions occur.

## Data Availability

The data used to support the findings of this study are included within the article.

## Supplementary Materials

The following supporting information can be downloaded at: https://www.mdpi.com/article/10.3390/antiox12111960/s1, Table S1: Climatic conditions recorded in the artichoke field on 29 and 30 January.
