# Supplementary material for: Influence of Artichoke Antioxidant Activity in Their Susceptibility to Suffer Frost Injury
Source: Antioxidants (Basel). 2023 Nov 2;12(11):1960. doi: 10.3390/antiox12111960 (PMC10669309; doi:10.3390/antiox12111960)
Supplement: Supplementary file 1 [file antioxidants-12-01960-s001.zip › antioxidants-2644888-supplementary.pdf]

Influence of artichoke antioxidant activity in their susceptibility to suffer frost injury

Marina Giménez-Berenguer, María Gutiérrez-Pozo, Vicente Serna-Escolano, Pedro Javier Zapata* and María José Giménez

Department of Food Technology, Escuela Politécnica Superior de Orihuela, University Miguel Hernández, Ctra. Beniel km. 3.2, 03312, Alicante, Spain; marina.gimenezb@umh.es (M.G.-B.); maria.gutierrezp@umh.es (M.G.-P.); vserna@umh.es (V.S.-E.); pedrojzapata@umh.es (P.J.Z.); maria.gimenezt@umh.es (M.J.G.).
* Correspondence: maria.gimenezt@umh.es

Supplementary Material

Table S1. Climatic conditions recorded in the artichoke field on the 29th and 30th of January.

**DAY**	**29th**				**30th**			
**HOUR**	**Temperature (ºC)**	**Relative Humidity (%)**	**WIND** Direction (tens of degrees)	**WIND** Speed (km/h)	**Temperature (ºC)**	**Relative Humidity (%)**	**WIND** Direction (tens of degrees)	**WIND** Speed (km/h)
**0**	2.2	82	-	0	6.4	76	19	2
**1**	1.9	85	-	0	4.8	78	-	0
**2**	1.3	81	-	0	4.1	83	23	2
**3**	2	83	-	0	3.1	83	-	0
**4**	0.9	84	25	2	2.1	85	-	0
**5**	0.9	79	24	3	1.3	85	-	0
**6**	-0.1	79	-	0	0.6	87	-	0
**7**	-1.3	83	-	0	-0.2	90	-	0
**8**	0.8	75	22	3	0.7	91	-	0
**9**	3.5	72	24	5	4.8	84	27	4
**10**	7.1	52	23	9	8	67	22	4
**11**	9.3	47	21	6	10.6	56	22	5
**12**	11.2	46	22	6	12.4	47	22	8
**13**	12.7	44	22	4	13.9	43	14	4
**14**	13.1	42	9	8	13.9	41	15	5
**15**	11.8	56	7	13	13.4	37	21	4

**16**	10.7	59	7	14	13.9	36	26	5
**17**	9.7	63	7	13	13.9	43	17	3
**18**	9.1	67	5	11	10.2	54	-	0
**19**	8.5	68	6	10	8.5	60	-	0
**20**	7.7	75	5	8	6.1	68	-	0
**21**	7.9	73	7	8	4.7	74	-	0
**22**	8.3	68	8	6	3.8	77	22	4
**23**	7.3	72	23	2	2.4	77	24	3
